# Impact of short-term air pollution exposure on premature rupture of membranes in the North China Plain, 2015–2021: a multicity case-crossover study

**DOI:** 10.7189/jogh.15.04165

**Published:** 2025-06-13

**Authors:** Na Wan, Zhenzhen Li, Zihan Zhang, Sisi Jiang, Hui Luo, Jianmiao Mo, Yinfeng Chen, Xiaolei Ma, Yuqing Zhang, Rongxiang Li, Heng He, Xiuhong Fu, Weihong Qiu

**Affiliations:** 1 Henan Key Laboratory of Fertility Protection and Aristogenesis, Luohe Central Hospital, Luohe, Henan, China; 2 Department of Epidemiology and Health Statistics, Fujian Provincial Key Laboratory of Environment Factors and Cancer, School of Public Health, Fujian Medical University, Fuzhou, China; 3 Institute of Population Medicine, Fujian Medical University, Fuzhou, Fujian, China

## Abstract

**Background:**

The effects of short-term air pollution exposure on hospital admissions for premature rupture of membranes (PROM) were still controversial.

**Methods:**

Daily data on PROM hospitalisations and air pollutants were collected from 1 January 2015 to 31 December 2021, across five cities in the North China Plain. We investigated the associations between short-term (lag0-7) exposure to air pollutants, including fine particulate matter (PM_2.5_), inhalable particulate matter (PM_10_), nitrogen dioxide (NO_2_), sulfur dioxide (SO_2_), carbon monoxide (CO), and ozone (O_3_), as well as composite air pollution indicators, including air quality index (AQI) and air quality composite index (AQCI). We further assessed the modifying effects of age (<35 and ≥35 years), season (cold and warm seasons), and the ‘Three-Year Action Plan’ (before and after implementation) on the above associations.

**Results:**

A total of 16 503 mothers with PROM were included. For each interquartile range (IQR) increase, the strongest relative risks (RRs) were 1.024 (95% confidence interval (CI) = 1.001–1.047, *P* < 0.05) in PM_2.5_ in lag3 (IQR = 48 μg/m^3^), RR = 1.036 (95% CI = 1.009–1.063, *P* < 0.05) in CO in lag4 (IQR = 0.49 mg/m^3^), RR = 1.024 (95% CI = 1.003–1.044, *P* < 0.05) in AQI in lag3 (IQR = 53), and RR = 1.026 (95% CI = 1.001–1.050, *P* < 0.05) in AQCI in lag3 (IQR = 2.9). The effects of exposure to PM_2.5_, NO_2_, SO_2_, CO, AQI, and AQCI on PROM were stronger in mothers aged <35 years, and the effects of exposure to PM_10_, NO_2_, and SO_2_ on PROM were stronger during the warm season (all *P*_modification_<0.05).

**Conclusions:**

Short-term air pollution exposure was related to elevated hospital risk of PROM. The effects were more pronounced among mothers aged <35 years and during the warm season.

Chinese people's living standards have greatly increased as a result of economic and social successes in recent decades. However, these accomplishments have also led to an increase in various environmental issues, including air pollution (smog, acid rain, *etc*.), water pollution (water eutrophication, industrial wastewater pollution, *etc*.), and soil pollution (heavy metal pollution, chemical pesticide pollution, *etc*.), which brings about series economic losses and pose serious health threats to the Chinese population [1,2]. In comparison with soil and water, contaminants in ambient air are more pervasively and closely related to human health. According to a report by the World Health Organization (WHO) [3], approximately 99% of the global population is exposed to air quality that exceeds the recommended limits established by the WHO, and air pollution is a major contributor to over seven million premature deaths annually around the world, with China accounting for approximately 1.8 million. The North China Plain (NCP) is located in the northern region of China and is the second-largest alluvial plain in China, with an area of about 300 000 square kilometres (km^2^) and a population of about 340 million. As an important industrial area in China, NCP is facing severe air pollution challenges. One recent study [4] reveals that fine particulate matter (PM_2.5_) concentration in early 2023 surged by 23 micrograms per cubic metre (μg/m^3^) compared to 2022, with 59% attributed to emission rebounds from industrial, power generation, and transportation sectors post-pandemic recovery.

Air pollutants can penetrate the circulatory system through the respiratory tract, affecting multiple organs and elevating the risk of numerous disorders, such as chronic obstructive pulmonary disease, asthma, stroke, and pregnancy-associated complications [5–7]. Premature rupture of membranes (PROM) is a natural rupture of membranes before labour, which is one of the most common complications in the perinatal period and can increase the rate of intrauterine infection, premature birth, and even perinatal mortality [8]. Premature rupture of membranes is categorised into two distinct categories: term PROM (tPROM), which occurs after 37 weeks of gestation, and preterm PROM (pPROM), which occurs before 37 weeks of gestation. The prevalence of tPROM among term pregnancies ranges from 8 to 10% [9], while the prevalence of pPROM among all pregnancies and preterm pregnancies is 2 to 3% and ~ 33% [10], respectively. Recent studies have investigated the association between acute exposure to air pollution and the risk of PROM, but the findings remain conflicting. For example, a spatiotemporal study in Spain [11] found that each interquartile range (IQR) increase in short-term exposure to nitrogen dioxide (NO_2_), nitrogen oxide (NO), and PM_2.5_ absorbance was associated with 42, 37, and 50% increase in the risk of PROM in case-control analyses, respectively, and exposure to PM_2.5_ absorbance (per IQR increase) was associated with a 47% increased risk in cohort analyses. A retrospective cohort study in the USA [12] found that short-term exposure to PM_2.5_ and ozone (O_3_) had a negative influence on PROM. Nonetheless, another study among 7121 singleton births in the USA [13] suggests that there is not enough evidence to support the adverse effect of short-term PM_2.5_ exposure on PROM. Similarly, several studies conducted in four cities in China, that is, Shanghai [14], Wuhan [15], Hefei [16,17], and Xinxiang [18], are also inconsistent due to huge variances in population characteristics as well as air pollution levels and components across China.

Air quality index (AQI) and air quality composite index (AQCI) serve as critical metrics for evaluating comprehensive air pollution, with distinct methodologies and applications. The AQI quantifies six criteria pollutants (PM_2.5_, inhalable particulate matter (PM_10_), NO_2_, sulphur dioxide (SO_2_), carbon monoxide (CO), O_3_) through a piecewise linear interpolation method to calculate individual sub-indices (IAQI), where the maximum IAQI determines the final AQI value [7]. However, AQI is unable to reflect multi-pollutant synergies, such as PM_2.5_-O_3_ co-pollution prevalent in regions like the NCP [19]. In contrast, the AQCI employs weighted aggregation to integrate cumulative health risks from concurrent pollutants, making it superior for analysing complex pollution scenarios [20]. Together, the two indices form a dual-layer framework: AQI for immediate public communication and AQCI for systemic pollution management, essential for addressing China's ‘dual high’ (PM_2.5_-O_3_) challenges under climatic and anthropogenic pressures.

Therefore, we selected five cities with high levels of both air pollution and population density in the NCP (Luohe, Pingdingshan, Xuchang, Zhoukou, and Zhumadian) to investigate the short-term effects of exposure to air pollutants (PM_2.5_, PM_10_, NO_2_, SO_2_, CO, and O_3_) on hospital admissions for PROM. In addition, we calculated two composite indicators of air pollution (AQI and AQCI) to reflect the overall levels of air pollution in the five cities and explore their associations with PROM hospitalisations. On this basis, we further investigate potential susceptible characteristics.

## METHODS

### Hospital admissions for PROM

We incorporated inpatients primarily diagnosed with PROM from five hospitals: Luohe Central Hospital, Pingdingshan Women and Child Health Care Hospital, Xuchang Central Hospital, Zhoukou Central Hospital, and Zhumadian Central Hospital, which have been previously described in detail [21,22]. The five hospitals are all well-known local hospitals that handle a large percentage of maternity work. According to the International Classification of Diseases (ICD-10), PROM is coded O42 and is diagnosed by obstetrician-gynaecologists from each hospital. The daily hospital admissions data of patients with PROM from 1 January 2015 to 31 December 2021 (a total of 2557 days) were collected from the hospital information system of the above hospitals.

The study protocol was approved by the Ethics Review Committee of Fujian Medical University (No. 2024-301).

### Air pollution and meteorological variables

The daily data on air pollutants and meteorological variables in the five cities were sourced from the real-time air quality monitoring stations established by the China National Environmental Monitoring Centre [23,24], as well as from the China Meteorological Data Sharing Service Centre [25]. The former included the daily average concentrations of PM_2.5_, PM_10_, NO_2_, SO_2_, and CO, and the peak 8-hour average concentration of O_3_. The latter included the daily average temperature (T) and relative humidity (RH). The AQI and AQCI were adopted to assess the overall level of air pollution in the five cities. The calculation methods for the two indices have been detailed in previous research [20,26], respectively.

### Statistical analyses

Descriptive statistical analyses were conducted to summarise the distributions of the daily number of PROM hospitalisations and levels of air pollution and meteorological variables across the five cities. The Spearman correlation coefficient was calculated to assess the relationships among the six individual air pollutants (PM_2.5_, PM_10_, NO_2_, SO_2_, CO, and O_3_), two composite air pollution indicators (AQI and AQCI), and two meteorological variables (T and RH), respectively.

A time-stratified case-crossover design was adopted in the study. In the design, the case day is defined as the day of hospitalisation, and the control days are systematically chosen from all non-case weeks within the same calendar month and the same day of week of the case day, ensuring that each control day was matched to the corresponding hospitalisation day by year, month, and day of week, both before and after the hospitalisation event [21,22]. Each case has 3–4 self-controls. For example, if a mother with PROM is hospitalised on 15 January 2015 (Thursday, case day), the days with the same month and day of week are selected as the control days, which are 1, 8, 22, and 29 January 2015.

Two stages of statistical analyses were performed in the study, which are described previously [21,22]. Due to the small probability of daily PROM hospitalisations, it is considered an approximate Poisson distribution. Therefore, we first employed a time-stratified case-crossover design with a quasi-Poisson generalised linear model to evaluate the associations between short-term (lag0-lag7) air pollution variables (including six individual air pollutants and two composite air pollution indicators) exposure and PROM hospitalisations in the five cities. In the model, each pollution variable was included sequentially; lag0 meant the air pollution levels on the day of hospital admission, lag1 meant the levels on the previous 1st day, and so on. Then, we included public holidays (binary variable), including New Year's Day, Spring Festival, Tomb-sweeping Day, Labour Day, Loong Boat Festival, Mid-Autumn Festival, and National Day, to adjust for the fluctuation of daily hospital admissions for PROM between holidays and nonholidays. Also, we included the T and RH on the day of hospitalisation to reduce their influence on air pollution factors and/or PROM [27]. In the second stage, we extracted the estimates and their standard error values of each city and conducted a random-effects meta-analysis using the ‘metafor’ package to aggregate the estimated effects of each city. The core model was as follows:

*logE (Y_t_) = β (AP) + stratum + public holidays + T + RH + intercept, family = quasiPoisson)* (1)

Where *E(Y_t_)* represents the estimated daily hospitalisations for PROM on day *t*. β represents the logarithmically (*log*) transformed relative risk (RR) along with the corresponding 95% confidence intervals (CI) for hospital admissions for PROM related to each IQR increment in air pollution variables. *AP* represents the six individual air pollutants (PM_2.5_, PM_10_, NO_2_, SO_2_, CO, and O_3_) or two composite air pollution indicators (AQI and AQCI). *Stratum* represents categorical time stratification in the analyses. Due to the discrete distribution of daily PROM hospitalisations, we chose a quasi-Poisson distribution as the connection function to control the over-dispersion of daily PROM hospitalisations.

Furthermore, to investigate the modifying effects of maternal age and seasonal variations [28], the data were stratified into distinct age groups (<35 and ≥35 years) and seasonal groups: the cold season (from November to April) and the warm season (from May to October). On 27 June 2018, the State Council of China announced the ‘Three-Year Action Plan for Winning the Blue-Sky Defense War’ aimed at mitigating the aggregate emission of significant air pollutants and greenhouse gases [29]. Therefore, we also compared the impact of the Action Plan (before and after implementation) on the above effects. Following that, stratified analyses were conducted, utilising a previously reported method [30] to determine significant differences among subgroups, as follows:



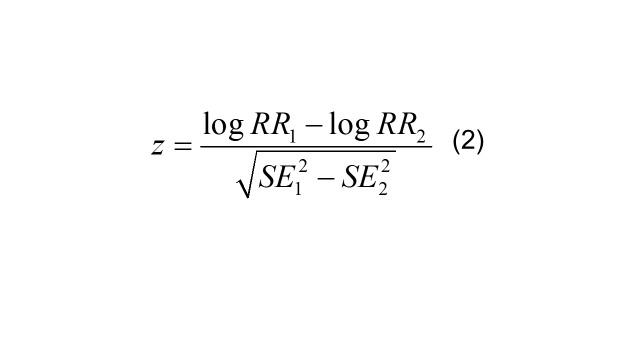

(2)


where *RR_1_* and *RR_2_* are the stratum-specific coefficients of point estimates, and SE*_1_* and SE*_2_* are the corresponding standard errors; *Z* is the Z-score, and the corresponding *P*-value is calculated based on statistical software.

### Sensitivity analyses

To ensure the robustness of our findings, we conducted sensitivity analyses by establishing multiple-pollutant models that adjusted for other air pollutants exhibiting Spearman's rank correlation coefficients (absolute values) less than 0.7. Additionally, we excluded each city in turn (leave-one-out) from the analyses to further validate our results.

All analyses were conducted using *R*, version 4.4.3 (R Foundation, Vienna, Austria). All statistical analyses were conducted with a two-sided approach with a significance level of 0.05.

## RESULTS

### Descriptive statistics

A total of 16 503 mothers with PROM were included, distributed as follows: 3125 (18.94%) from LuoHe Central Hospital, 6635 (40.20%) from Pingdingshan Women and Child Health Care Hospital, 1287 (7.80%) from Xuchang Central Hospital, 2308 (13.99%) from Zhoukou Central Hospital, and 3148 (19.08%) from Zhumadian Central Hospital. The average age of participants was 29.22 ± 5.05 years, with 84.77% of them aged <35 years. The majority were married (97.85%) and were farmers or unemployed (58.06%). The characteristics of the participants across the five cities were similar (**Table 1**).

**Table 1:** Characteristics of participants with PROM in the five Chinese cities, 2015–2021

Characteristics	Total	Luohe	Pingdingshan	Xuchang	Zhoukou	Zhumadian
PROM, n (%)	16 503 (100.00)	3125 (18.94)	6635 (40.20)	1287 (7.80)	2308 (13.99)	3148 (19.08)
Age, years (mean ± SD)	29.22 ± 5.05	29.47 ± 4.70	29.47 ± 4.97	29.73 ± 4.96	28.36 ± 5.17	28.85 ± 5.38
Age group (%)						
*<35 y*	13 989 (84.77)	2645 (84.64)	5623 (84.75)	1064 (82.67)	2019 (87.48)	2638 (83.80)
*≥35 y*	2513 (15.23)	480 (15.36)	1012 (15.25)	223 (17.33)	289 (12.52)	509 (16.17)
*Missing*	1 (0.01)	0 (0.00)	0 (0.00)	0 (0.00)	0 (0.00)	1 (0.03)
Marital status, n (%)*						
*Not married*	212 (1.49)	44 (1.41)	101 (1.52)	31 (2.41)	\	36 (1.14)
*Married*	13 890 (97.85)	3026 (96.83)	6512 (98.15)	1251 (97.20)	\	3101 (98.51)
*Divorced/widowed*	28 (0.20)	11 (0.35)	9 (0.14)	4 (0.31)	\	4 (0.13)
*Missing*	65 (0.46)	44 (1.41)	13 (0.20)	1 (0.08)	\	7 (0.22)
Occupation, n (%)						
*Farmer or unemployment*	9581 (58.06)	976 (31.23)	4282 (64.54)	671 (52.14)	1642 (71.14)	2010 (63.85)
*Worker*	656 (3.98)	109 (3.49)	481 (7.25)	14 (1.09)	4 (0.17)	48 (1.52)
*Sales or service*	643 (3.90)	315 (10.08)	83 (1.25)	54 (4.20)	109 (4.72)	82 (2.60)
*Office job*	4093 (24.80)	1042 (33.34)	1227 (18.49)	507 (39.39)	516 (22.36)	801 (25.44)
*Other*	1371 (8.31)	647 (20.70)	474 (7.14)	41 (3.19)	37 (1.60)	172 (5.46)
*Missing*	159 (0.96)	36 (1.15)	88 (1.33)	0 (0.00)	0 (0.00)	35 (1.11)

PROM – premature rupture of membranes, SD – standard deviation

*Included four cities except Zhoukou.

**Figure 1:**
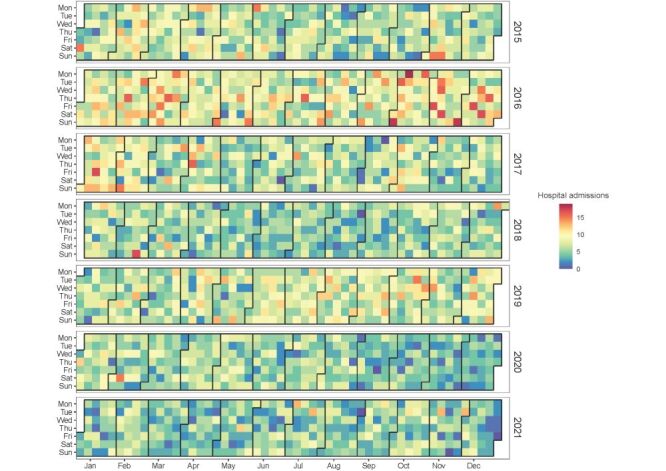
Combined time-series plots of daily premature rupture of membranes (PROM) hospitalisations in the five cities, 2015–2021.

We drew the daily distribution of the total number of PROM patients in five cities from 2015 to 2021 (**Figure 1**), as well as the distribution in each city (Figure S1 in the **Online Supplementary Document**). We were unable to observe any long-term trend in PROM hospital admissions (detailed results provided in Table S1 in the **Online Supplementary Document**). Table S2 in the **Online Supplementary Document** details the daily hospital admissions of PROM and the corresponding levels of air pollution and meteorological variables in the cities. The daily average number of participants with PROM was 6.45 ± 2.97 in these cities. The number of participants with PROM was higher under the age of 35, in the cold season, and before the implementation of the Action Plan, respectively.

### Distributions of air pollution and meteorological variables and their Spearman’s rank correlation coefficients

**Figure 2:**
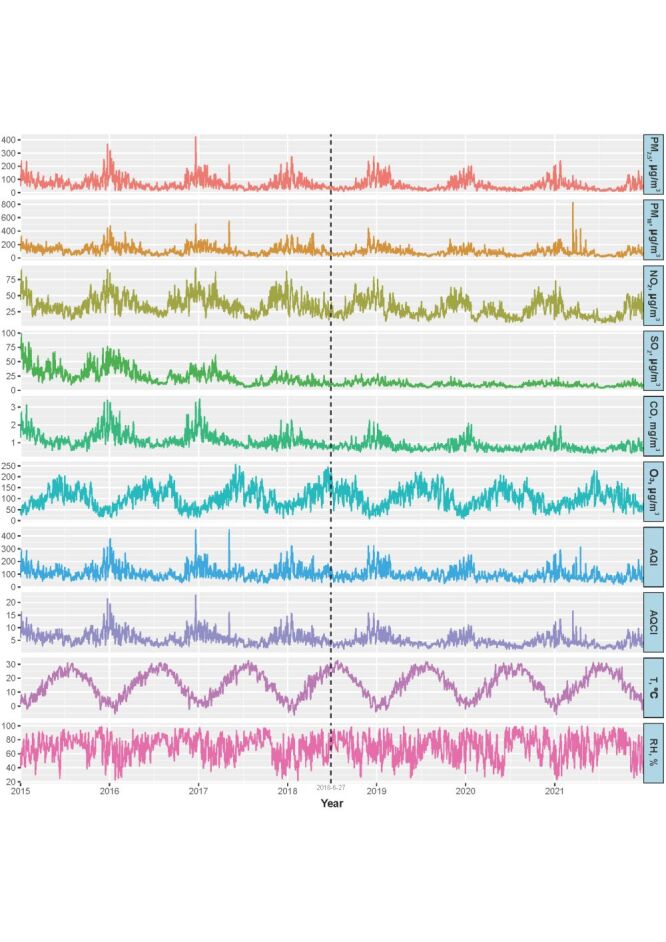
Combined time-series plots of daily air pollution and meteorological variables in the five cities, 2015–2021.

**Figure 2** depicts long-term trends in daily average air pollution and meteorological variables in five cities from 2015 to 2021 (detailed results provided in Table S1 in the **Online Supplementary Document**). Except for the O_3_, the levels of other air pollutants have decreased over the years, particularly SO_2_. The levels of all air pollution variables (except O_3_) were higher in the cold season compared to the warm season, whereas meteorological variables (T and RH) exhibited opposite trends.

The combined Spearman correlations among air pollution and meteorological variables in five cities from 2015 to 2021 are shown in Figure S2 in the **Online Supplementary Document**. High positive correlations (|r|≥0.7 and *P* < 0.05) were observed between PM_2.5_ and PM_10_/NO_2_/CO/AQI/AQCI, PM_10_ and NO_2_/AQI/AQCI, NO_2_ and SO_2_/AQCI, SO_2_ and AQCI, CO and AQCI, O_3_ and T, as well as AQI and AQCI. All other air pollutants except for O_3_ were highly positively correlated with AQCI (|r≥|0.7 and *P* < 0.05) while exhibiting low to moderate correlations with T and RH.

### Short-term effect of air pollution exposure on PROM hospital admissions

**Figure 3:**
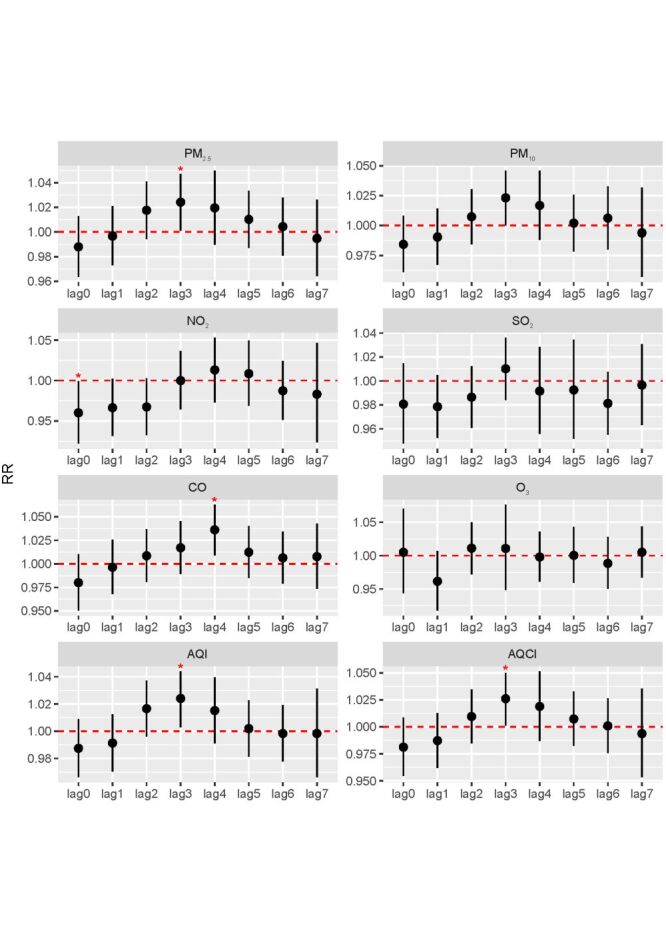
Combined associations of Short-term air pollution exposure with hospital admissions for premature rupture of membranes (PROM) in the five cities, 2015–2021.

We observe significant positive associations between exposure to PM_2.5_, CO, AQI, and AQCI with hospital admissions for PROM during a 7-day exposure window (lag0-7) among the five cities (**Figure 3**). Specifically, for each IQR increase, we found the strongest RR = 1.024 (95% CI = 1.001–1.047, *P* < 0.05) for PM_2.5_ in lag3 (IQR = 48 μg/m^3^), RR = 1.036 (95% CI = 1.009–1.063, *P* < 0.05) for CO in lag4 (IQR = 0.49 mgmes per cubic metre (mg/m^3^)), RR = 1.024 (95% CI = 1.003–1.044, *P* < 0.05) for AQI in lag3 (IQR = 53), and RR = 1.026 (95% CI = 1.001–1.050, *P* < 0.05) for AQCI in lag3 (IQR = 2.9). The city-specific and overall effects on the days with the strongest effect estimates are presented in Figure S3 in the **Online Supplementary Document**.

Stratified analyses were conducted to assess the potential modifying effects of maternal age, season, and the Action Plan on the associations, and the results are shown in Figure S4–6 in the **Online Supplementary Document**, respectively. Compared to mothers aged ≥35 years, the effects of exposure to PM_2.5_, NO_2_, SO_2_, CO, AQI, and AQCI on PROM in lag4 were more pronounced in mothers aged <35 years (all *P*_modification_<0.05) (Figure S4 in the **Online Supplementary Document**). The effects of PM_10_ (lag2), NO_2_ (lag0), and SO_2_ (lag0) on PROM were more pronounced during the warm season than during the cold season (all *P*_modification_<0.05) (Figure S5 in the **Online Supplementary Document**). For the Action Plan, we found significant positive associations between PM_10_ (lag3) and AQI (lag3) with PROM in the period before the implementation of the Action Plan, but no significant modifying effect was observed (Figure S6 in the **Online Supplementary Document**).

### Sensitivity analyses

Due to the strong collinearity between air pollutants, we included other air pollutants with Spearman rank correlation coefficients (absolute values) less than 0.7 with the target pollutant in a two-pollutant model to control the impact of other pollutants on PROM (**Table 2**). The results reveal that the effect estimates of the primary pollutants remained robust after adjusting for coexisting air pollutants in two-pollutant models, with no statistically significant differences observed compared to single-pollutant models. Further sensitivity analyses were performed by sequentially excluding subsamples from each city to evaluate the geographic robustness of our findings. The results indicated that the association strength between air pollution variables and PROM remained relatively stable (Table S3 in the **Online Supplementary Document**).

**Table 2:** Relative risks of PROM with an IQR increase in air pollutants using single- and two-pollutant models

Pollutants models*	RR	95% CI	*P-*value
**PM_2.5_**			
Single pollutant model	1.024	1.001–1.047	0.045
*+ SO_2_*	1.024	1.000–1.049	0.046
*+ O_3_*	1.024	1.001–1.047	0.045
**PM_10_**			
Single pollutant model	1.023	1.000–1.046	0.051
*+ SO_2_*	1.024	1.000–1.049	0.054
*+ CO*	1.015	0.990–1.039	0.239
*+ O_3_*	1.023	1.000–1.046	0.053
**NO_2_**			
Single pollutant model	1.013	0.973–1.053	0.551
*+ CO*	0.979	0.922–1.039	0.481
*+ O_3_*	1.011	0.970–1.053	0.614
**SO_2_**			
Single pollutant model	1.010	0.984–1.036	0.449
*+ PM_2.5_*	1.004	0.977–1.031	0.776
*+ PM_10_*	1.003	0.976–1.031	0.830
*+ CO*	1.010	0.984–1.037	0.457
*+ O_3_*	1.009	0.983–1.036	0.487
**CO**			
Single pollutant model	1.036	1.009–1.063	0.012
*+ PM_10_*	1.030	1.001–1.059	0.045
*+ NO_2_*	1.040	1.006–1.075	0.020
*+ SO_2_*	1.035	1.007–1.063	0.014
*+ O_3_*	1.035	1.008–1.064	0.011
**O_3_**			
Single pollutant model	1.011	0.972–1.050	0.587
*+ PM_2.5_*	1.011	0.972–1.051	0.598
*+ PM_10_*	1.009	0.970–1.049	0.659
*+ NO_2_*	1.010	0.972–1.051	0.605
*+ SO_2_*	1.011	0.972–1.052	0.585
*+ CO*	1.012	0.974–1.053	0.534

CI – confidence interval, CO – carbon monoxide, IQR – interquartile range, NO_2_ – nitrogen dioxide, O_3_ – ozone, PM_2.5_ – fine particulate matter, PM_10_ – inhalable particulate matter, RR – relative risk, PROM – premature rupture of membranes, SO_2_ – sulfur dioxide

*PM_2.5_, lag3 d; PM_10_, lag3 d; NO_2_, lag4 d; SO_2_, lag3 d; CO, lag4 d; O_3_, lag2 d.

## DISCUSSION

In this study, we assessed the short-term effects of exposure to six air pollutants (PM_2.5_, PM_10_, NO_2_, SO_2_, CO, and O_3_) and two composite air pollution indicators (AQI and AQCI) on hospital admissions for PROM in five higher-polluted cities in the North China Plain. Our findings reveal that short-term exposure to PM_2.5_ (lag3) and CO (lag4), as well as AQI (lag3) and AQCI (lag3), is positively associated with hospitalisation for PROM. These associations can be modified by maternal age and season. Specifically, the effects of exposure to PM_2.5_, NO_2_, SO_2_, CO, AQI, and AQCI are more pronounced among mothers aged <35 years, and the effects of exposure to PM_10_, NO_2_, and SO_2_ are stronger during the warm season.

As a principal component of ambient air pollutants, short-term PM_2.5_ exposure demonstrates epidemiological associations with PROM. A time-series study [14] in Shanghai, China (n = 3133 pPROM cases; mean maternal age = 31.10) identified elevated pPROM risks per 10 μg/m^3^ PM_2.5_ increase at lag2-lag3 (mean (x̄) = 44.87, standard deviation (SD) concentration = 32.46 μg/m^3^), with the maximum excess risk of 13.06% (95% CI = 2.66–24.52) at lag3. This aligns with another Chinese study [31] that enrolled 45 879 singleton births, showing PM_2.5_ exposure (x̄ = 51.11, SD concentration = 36.56 μg/m^3^) is a risk for PROM and pPROM. Similarly, a retrospective cohort study in the USA [12] (n = 15 588 PROM cases; mean age = 28.1) demonstrated a 3–4% increased PROM incidence per IQR PM_2.5_ (x̄ = 9.1, SD concentration = 8.9 μg/m^3^) increase during the five-hour pre-delivery window. However, contradictory evidence emerges from other geographical contexts. For example, a time-series analysis in Hefei, China [16] (n = 6899 singleton pregnancies) found no significant PM_2.5_-PROM association despite comparable exposure levels (x̄ = 54.58, SD concentration = 32.33 μg/m^3^) to our study. Similarly, another study in Barcelona, Spain [11] enrolled 5555 mothers (median age = 30) also failed to detect any significant association during the final trimester of pregnancy between PM_2.5_ exposure and pPROM in both matched case-control and cohort analyses (entire pregnancy median (IQR) concentration = 19.8 (4.1) μg/m^3^). These discrepancies may stem from three methodological and contextual factors [32–34]:

1) Regional heterogeneity in population susceptibility profiles (*e.g*. genetic predispositions, lifestyles, comorbidities);

2) Regional differences in PM_2.5_ chemical composition and emission levels;

3) Methodological differences in exposure window definitions (acute *vs*. cumulative lags) and assessment protocols (fixed-site monitoring *vs*. spatiotemporal models).

Future research should prioritise toxicity analysis and sensitive biomarkers specific to PM_2.5_ components, further elucidate the biological mechanisms, and identify high-risk subgroups.

We also found a positive association between short-term CO exposure and PROM. Carbon monoxide is an odourless, colourless, and non-irritating gas, primarily generated through the incomplete combustion of carbonaceous substances [35]. Beyond PROM [17], short-term CO exposure can induce various adverse pregnancy outcomes, including preterm birth [36], intrauterine growth retardation, and reduced birth weight [37]. Nevertheless, there are still several studies that have not identified any association between short-term CO exposure and PROM [12], indicating the need for further epidemiological and/or toxicological studies to elucidate the association between CO exposure and PROM. Despite the recent enforcement of China's stringent air quality improvement plan [29], the present study still uncovered significant adverse effects of two composite air pollution indicators, AQI and AQCI, on PROM. This suggests the imperative for the government and society to adopt more rigorous air pollution control measures and persistently tackle the adverse effects of overall air pollution on the health of pregnant women.

Despite the absence of a definitive biological mechanism to explain the association between air pollution and PROM, extant evidence suggests that inflammatory response and oxidative stress may represent the primary pathways [38]. Foetal membrane integrity relies on extracellular matrix composition, particularly collagen and elastin [39]. Progressive declines in collagen-synthesising amniotic mesenchymal cell density and catalytic enzyme activity across gestation may render membranes increasingly vulnerable to pollutant-induced damage [40]. PM_2.5_, characterised by a small aerodynamic diameter and a relatively large specific surface area, can adsorb various toxic and harmful substances in the environment, such as heavy metals and volatile organic pollutants [21,22]. These pollutants can enter the maternal body and traverse placental tissues, accumulating on the foetal side [41]. One animal study [42] demonstrates that PM_2.5_ exposure during both pre-pregnancy and gestational periods in rats significantly elevates interleukin-4 levels in the foetal side of the placenta, leading to intrauterine inflammation. PM, as a potent oxidising agent, catalyses the generation of reactive oxygen species, inducing mitochondrial dysfunction and DNA damage in placental tissues [43]. The resultant inflammatory and oxidative stress responses may degrade collagen within the foetal membranes, compromising their tensile strength and increasing susceptibility to premature rupture [31,44]. In addition, CO has a strong affinity for haemoglobin (230–270 times higher than oxygen), which can form carboxyhaemoglobin, leading to a decrease in haemoglobin's oxygen-carrying capacity and affecting the dissociation of oxygenated haemoglobin, which reduces maternal oxygen-carrying capacity and induces placental hypoxia [37,45]. Notably, iron supplementation may mitigate CO-associated risks of premature membrane rupture by enhancing haemoglobin-oxygen affinity, thereby improving oxygen delivery to placental tissues [17].

Our subgroup analyses showed that the deleterious effects of air pollution exposure on PROM were more pronounced in younger mothers. Our findings are similar to Zhang et al. [16], who observed that the effects of NO_2_ and SO_2_ on PROM hospitalisations were more significant among younger mother groups (<35 years old). The organs and systems of younger pregnant women, particularly their reproductive organs and pelvis, are not yet fully developed, potentially compromising their capacity to withstand the physiological demands of pregnancy and childbirth, which may elevate the risks of premature birth, low birth weight, and malnutrition [46]. Given the seasonal variations in air pollution, we employed stratified analyses to assess the modifying effects of seasonality on our findings. Our results revealed that the effects were more pronounced during the warm season compared to the cold season. A similar study in Xinxiang, China, found stronger O_3_ effects on PROM in the warm season [18]. Another study conducted in Hefei, China, also demonstrated stronger effects of NO_2_ during the warm season [16]. However, they found that the stronger effects of SO_2_ on PROM hospitalisations occurred in the cold season, which is the opposite of ours. There are two possible reasons:

1) With the strict implementation of China's air improvement programme, the concentrations of air pollutants have decreased year by year, especially SO_2_ and PM, which leads to the reduction of the difference between warm and cold seasons.

2) Due to the less of outdoor activities of pregnant women in the cold season, the individual exposure level to air pollution decreased to a certain extent.

Our research has several advantages. First, we comprehensively assess the impacts of six major air pollutants and two composite air pollution indicators on the risk of PROM hospitalisations, utilising data from five cities with severe air pollution in the North China Plain. Furthermore, we conducted sensitivity analyses by establishing multi-pollutant models and excluding each city in turn (leave-one-out) to ensure the robustness of our findings. However, there are some limitations to this study. First, due to the absence of individual-level exposure information, we used daily city-level average concentrations of air pollutants to represent individual levels, which may introduce exposure assessment errors. Additionally, our research belongs to an ecological study. There may be ecological fallacies, and it is difficult to judge the causal relationship between exposure and outcomes. Our findings require further validation through comprehensive epidemiological and toxicological research.

## CONCLUSIONS

Short-term air pollution exposure may increase the risk of premature rupture of membranes during hospitalisation. These effects are more obvious in mothers under 35 and during the warm season. Our results show that it is necessary to continue to control air pollution and provide targeted protective measures for pregnant women, such as reducing going out in polluted weather or doing well in going out protection, using indoor air purification equipment, and opening windows for ventilation when the air quality is good.

## Additional material


Online Supplementary Document
