# Supplementary material for: Impact of short-term air pollution exposure on premature rupture of membranes in the North China Plain, 2015–2021: a multicity case-crossover study
Source: J Glob Health. 2025 Jun 13;15:04165. doi: 10.7189/jogh.15.04165 (PMC12163860; doi:10.7189/jogh.15.04165)

Supplement to: Wan N, Li Z, Zhang Z, Jiang S, Luo H, Mo J, Chen Y, Ma X, Zhang Y, Li R, He H, Fu X, Qiu W. Impact of short-term air pollution exposure on premature rupture of membranes in the North China Plain, 2015–2021: a multicity case-crossover study. J Glob Health. 2025;15:04165.

Table S1. Summary descriptive statistics on the daily hospital admissions of PROM and levels of air pollution and meteorological factors from 2015 to 2021.

Factors	2015		2016		2017		2018		2019		2020		2021	
	mean (SD)	median (IQR)	mean (SD)	median (IQR)	mean (SD)	median (IQR)	mean (SD)	median (IQR)	mean (SD)	median (IQR)	mean (SD)	median (IQR)	mean (SD)	median (IQR)
PROM	6.69±2.74	7 (3)	8.73±3.21	8 (5)	6.75±2.99	6 (5)	5.52±2.41	5 (3)	7.23±2.75	7 (4)	5.21±2.30	5 (3)	5.04 ± 2.47	5 (4)
$PM_{2.5}$, μg/m^3	81.4 ± 42.5	74.4 (46.8)	70.8 ± 53.3	53.5 (48.7)	63.1 ± 41.0	51.2 (42.2)	61.8 ± 44.3	46.8 (37.8)	57.6 ± 42.0	41.6 (45.2)	51.0 ± 35.7	39.3 (34.4)	46.0 ± 36.0	35.2 (31.8)
PM_{10}, μg/m^3	132.8 ± 55.4	129.0 (68.8)	121.7 ± 74.0	98.5 (88.9)	111.9 ± 63.2	99.2 (62.4)	111.7 ± 68.5	91.2 (68.6)	95.0 ± 50.1	81.0 (62.4)	80.1 ± 40.7	73.5 (54.3)	83.5 ± 69.8	68.6 (56.6)
NO_2, μg/m^3	36.4 ± 13.9	34.0 (17.6)	39.0 ± 13.8	36.1 (19.4)	37.7 ± 14.9	35.0 (21.2)	34.2 ± 13.2	31.8 (17.8)	30.4 ± 12.1	27.6 (17.0)	26.7 ± 11.8	24.6 (17.6)	23.7 ± 11.9	20.6 (15.2)
SO_2, μg/m^3	34.9 ± 15.0	31.8 (16.4)	28.1 ± 13.9	24.0 (19.4)	19.4 ± 9.5	18.6 (12.8)	13.9 ± 4.8	13.0 (6.8)	10.8 ± 3.9	10.0 (4.6)	9.5 ± 3.1	9.2 (4.6)	8.9 ± 3.1	8.4 (3.6)
CO, mg/m^3	1.3 ± 0.5	1.2 (0.6)	1.3 ± 0.5	1.1 (0.5)	1.1 ± 0.4	1.0 (0.4)	0.9 ± 0.3	0.9 (0.3)	0.8 ± 0.3	0.8 (0.3)	0.8 ± 0.2	0.7 (0.2)	0.7 ± 0.2	0.7 (0.2)
O_3, μg/m^3	96.8 ± 42.9	94.4 (66.4)	99.9 ± 44.7	100.2 (73.9)	106.4 ± 47.3	99.0 (68.2)	109.6 ± 49.5	103.0 (73.0)	110.0 ± 48.8	109.6 (78.8)	101.9 ± 40.1	100.9 (64.0)	98.2 ± 37.4	91.0 (48.4)
AQI	115.8 ± 45.7	108.4 (53.4)	109.8 ± 56.8	95.1 (53.6)	105.9 ± 48.9	95.2 (47.8)	107.7 ± 50.4	95.0 (54.4)	104.3 ± 43.3	97.0 (48.2)	90.1 ± 38.0	83.1 (39.7)	85.5 ± 43.7	72.8 (41.8)

AQCI	6.6 ± 2.4	6.4 (2.7)	6.1 ± 3.0	5.3 (3.5)	5.6 ± 2.4	5.1 (2.5)	5.4 ± 2.4	4.8 (2.5)	4.8 ± 2.0	4.3 (2.2)	4.3 ± 1.7	4.0 (2.1)	4.0 ± 2.1	3.5 (2.2)
T, ° C	15.0 ± 9.1	16.1 (16.5)	15.5 ± 9.6	16.3 (17.4)	15.6 ± 9.4	15.8 (17.4)	15.6 ± 10.2	17.1 (18.2)	15.8 ± 9.9	16.5 (18.6)	15.7 ± 9.4	16.0 (17.8)	16.0 ± 8.9	15.1 (16.1)
RH, %	70.8 ± 14.2	71.6 (19.2)	71.1 ± 14.6	73.1 (20.0)	69.0 ± 15.6	70.8 (22.4)	68.4 ± 15.5	69.4 (24.2)	66.6 ± 15.8	67.6 (24.6)	70.6 ± 17.6	74.1 (29.3)	68.5 ± 17.8	72.0 (26.4)

Abbreviation: SD, standard deviation; IQR, interquartile range; PROM, premature rupture of membranes; $PM_{2.5}$, fine particulate matter; PM_{10}, inhalable particulate matter; NO_2, nitrogen dioxide; SO_2, sulfur dioxide; CO, carbon monoxide; O_3, ozone; AQI, air quality index; AQCI, air quality composite index; T, temperature; RH, relative humidity.

Table S2. Summary descriptive statistics on the daily hospital admissions of PROM and levels of air pollution and meteorological factors in the five Chinese cities.

Variables	Total mean ± SD	Total median (IQR)	Luohe mean ± SD	Luohe median (IQR)	Pingdingshan mean ± SD	Pingdingshan median (IQR)	Xuchang mean ± SD	Xuchang median (IQR)	Zhoukou mean ± SD	Zhoukou median (IQR)	Zhumadian mean ± SD	Zhumadian median (IQR)
Total	6.45 ± 2.97	6 (4)	1.22 ± 1.20	1 (2)	2.59 ± 1.77	2 (3)	0.50 ± 0.73	0 (1)	0.90 ± 1.11	1 (1)	1.23 ± 1.20	1 (2)
Age												
<35 years	5.47 ± 2.70	5 (4)	1.03 ± 1.10	1 (2)	2.20 ± 1.60	2 (2)	0.42 ± 0.66	0 (1)	0.79 ± 1.02	0 (1)	1.03 ± 1.09	1 (2)
≥35 years	0.98 ± 1.05	1 (2)	0.19 ± 0.44	0 (0)	0.40 ± 0.65	0 (1)	0.09 ± 0.30	0 (0)	0.11 ± 0.35	0 (0)	0.20 ± 0.46	0 (0)
Seasons												
Cold season	6.64 ± 3.07	6 (5)	1.27 ± 1.24	1 (2)	2.62 ± 1.80	2 (3)	0.51 ± 0.72	0 (1)	0.99 ± 1.20	1 (2)	1.25 ± 1.21	1 (2)
Warm season	6.27 ± 2.85	6 (4)	1.17 ± 1.16	1 (2)	2.57 ± 1.73	2 (3)	0.50 ± 0.74	0 (1)	0.82 ± 1.01	1 (1)	1.22 ± 1.18	1 (2)
The Action Plan												
Before	7.15 ± 3.11	7 (4)	1.31 ± 1.23	1 (2)	3.04 ± 1.86	3 (2)	0.64 ± 0.81	0 (1)	0.88 ± 1.17	1 (1)	1.27 ± 1.28	1 (2)
After	5.77 ± 2.65	6 (3)	1.13 ± 1.16	1 (2)	2.15 ± 1.55	2 (2)	0.37 ± 0.61	0 (1)	0.92 ± 1.05	1 (1)	1.19 ± 1.11	1 (2)
Air pollutants	6.45 ± 2.97	6 (4)	1.22 ± 1.20	1 (2)	2.59 ± 1.77	2 (3)	0.50 ± 0.73	0 (1)	0.90 ± 1.11	1 (1)	1.23 ± 1.20	1 (2)

$PM_{2.5}$, μg/m^3	61.7 ± 43.8	47.8 (47.4)	64.0 ± 47.8	49.0 (51.0)	64.5 ± 44.6	51.0 (48.0)	62.2 ± 48.0	47.0 (47.0)	60.6 ± 47.4	46.0 (49.0)	57.1 ± 40.6	45.0 (43.0)
PM_{10}, μg/m^3	105.2 ± 63.9	91.0 (70.6)	109.9 ± 70.3	94.0 (78.0)	109.5 ± 67.0	92.0 (75.0)	104.2 ± 67.2	89.0 (70.0)	103.1 ± 66.2	89.0 (71.0)	99.5 ± 61.7	85.0 (70.0)
NO_2, μg/m^3	32.6 ± 14.2	30.0 (19.8)	31.6 ± 15.4	29.0 (21.0)	36.5 ± 15.1	34.0 (21.0)	37.8 ± 17.7	34.0 (23.0)	26.9 ± 12.6	24.0 (17.0)	30.1 ± 15.2	27.0 (20.0)
SO_2, μg/m^3	17.9 ± 13.0	13.2 (13.4)	16.8 ± 13.8	12.0 (11.0)	22.7 ± 18.2	16.0 (17.0)	18.6 ± 14.5	14.0 (15.0)	13.9 ± 9.1	11.0 (9.0)	17.6 ± 18.1	11.0 (14.0)
CO, mg/m^3	1.0 ± 0.4	0.9 (0.4)	0.9 ± 0.4	0.8 (0.4)	1.0 ± 0.4	0.9 (0.4)	1.2 ± 0.5	1.0 (0.5)	1.1 ± 0.6	0.9 (0.5)	0.8 ± 0.4	0.7 (0.4)
O_3, μg/m^3	103.3 ± 44.8	99.2 (66.8)	104.2 ± 46.0	100.0 (68.0)	105.6 ± 47.5	100.0 (71.0)	101.3 ± 48.0	97.0 (70.0)	101.3 ± 45.1	97.0 (67.0)	104.0 ± 45.6	100.0 (64.0)
AQI	102.7 ± 48.0	92.2 (50.8)	105.5 ± 52.8	94.0 (55.0)	105.5 ± 51.1	94.0 (55.0)	103.8 ± 53.4	91.0 (54.0)	101.7 ± 51.0	90.0 (52.0)	97.1 ± 45.7	88.0 (49.0)
AQCI	5.3 ± 2.5	4.7 (2.9)	5.3 ± 2.7	4.8 (3.0)	5.6 ± 2.6	5.0 (3.1)	5.4 ± 2.7	4.8 (3.0)	5.0 ± 2.5	4.5 (2.8)	4.9 ± 2.4	4.4 (2.8)
Meteorological factors												
T, °C	15.6 ± 9.5	16.1 (17.3)	15.4 ± 9.4	16.0 (17.0)	15.8 ± 9.5	16.1 (17.4)	14.9 ± 9.7	15.5 (17.9)	16.3 ± 9.6	16.9 (17.2)	15.6 ± 9.3	16.2 (16.6)
RH, %	69.3 ± 16.0	71.0 (23.6)	68.6 ± 16.4	70.0 (24.0)	66.9 ± 17.6	69.0 (25.0)	72.1 ± 16.3	75.0 (23.0)	67.0 ± 17.1	68.0 (26.0)	71.8 ± 16.7	74.0 (25.0)

Abbreviation: SD, standard deviation; IQR, interquartile range; $PM_{2.5}$, fine particulate matter; PM_{10}, inhalable particulate matter; NO_2, nitrogen dioxide; SO_2, sulfur dioxide; CO, carbon monoxide; O_3, ozone; AQI, air quality index; AQCI, air quality composite index; T, temperature; RH, relative humidity.

Table S3. Relative risks and 95% confidence intervals of PROM with an IQR increase in air pollution variables ($PM_{2.5}$, PM_{10}, SO_2, NO_2, O_3, AQI, and AQCI) after excluding each city in turn.

Pollutants and models *	RR	95% CI	*P* value
Excluding Luohe			
$PM_{2.5}$	1.024	0.998 to 1.050	0.066
PM_{10}	1.025	1.000 to 1.051	0.049
NO_2	1.025	0.985 to 1.068	0.223
SO_2	1.012	0.984 to 1.040	0.416
CO	1.036	1.006 to 1.067	0.017
O_3	1.007	0.965 to 1.051	0.744
AQI	1.025	1.002 to 1.048	0.032
AQCI	1.028	1.000 to 1.056	0.048
Excluding Pingdingshan			
$PM_{2.5}$	1.023	0.993 to 1.054	0.129
PM_{10}	1.030	1.000 to 1.061	0.047
NO_2	0.986	0.939 to 1.035	0.561
SO_2	1.017	0.981 to 1.055	0.355
CO	1.033	0.998 to 1.069	0.063
O_3	1.018	0.968 to 1.071	0.486
AQI	1.026	0.999 to 1.054	0.060
AQCI	1.028	0.995 to 1.061	0.100
Excluding Xuchang			
$PM_{2.5}$	1.028	1.004 to 1.053	0.024
PM_{10}	1.022	0.998 to 1.047	0.077
NO_2	1.005	0.955 to 1.057	0.857
SO_2	1.013	0.985 to 1.041	0.371

CO	1.043	1.013 to 1.074	0.005
O_3	1.010	0.970 to 1.052	0.626
AQI	1.025	1.003 to 1.047	0.026
AQCI	1.028	1.001 to 1.055	0.039
Excluding Zhoukou			
$PM_{2.5}$	1.022	0.997 to 1.048	0.081
PM_{10}	1.020	0.996 to 1.046	0.109
NO_2	1.021	0.982 to 1.062	0.289
SO_2	1.012	0.985 to 1.040	0.373
CO	1.034	1.003 to 1.065	0.029
O_3	1.017	0.976 to 1.060	0.430
AQI	1.024	1.001 to 1.046	0.037
AQCI	1.025	0.998 to 1.052	0.071
Excluding Zhumadian			
$PM_{2.5}$	1.020	0.995 to 1.046	0.117
PM_{10}	1.019	0.994 to 1.044	0.145
NO_2	1.011	0.959 to 1.065	0.694
SO_2	0.998	0.969 to 1.028	0.903
CO	1.031	1.001 to 1.061	0.043
O_3	1.003	0.960 to 1.048	0.886
AQI	1.020	0.998 to 1.043	0.082
AQCI	1.020	0.993 to 1.048	0.150

Abbreviation: RR, relative risk; CI, confidence interval; $PM_{2.5}$, fine particulate matter; PM_{10}, inhalable particulate matter; NO_2, nitrogen dioxide; SO_2, sulfur dioxide; CO, carbon monoxide; O_3, ozone; AQI, air quality index; AQCI, air quality composite index.

* $PM_{2.5}$, lag3 day; PM_{10}, lag3 day; NO_2, lag4 day; SO2, lag3 day; CO, lag4 day; O_3, lag2 day; AQI, lag3 day; AQCI, lag3 day.

Figure S1.

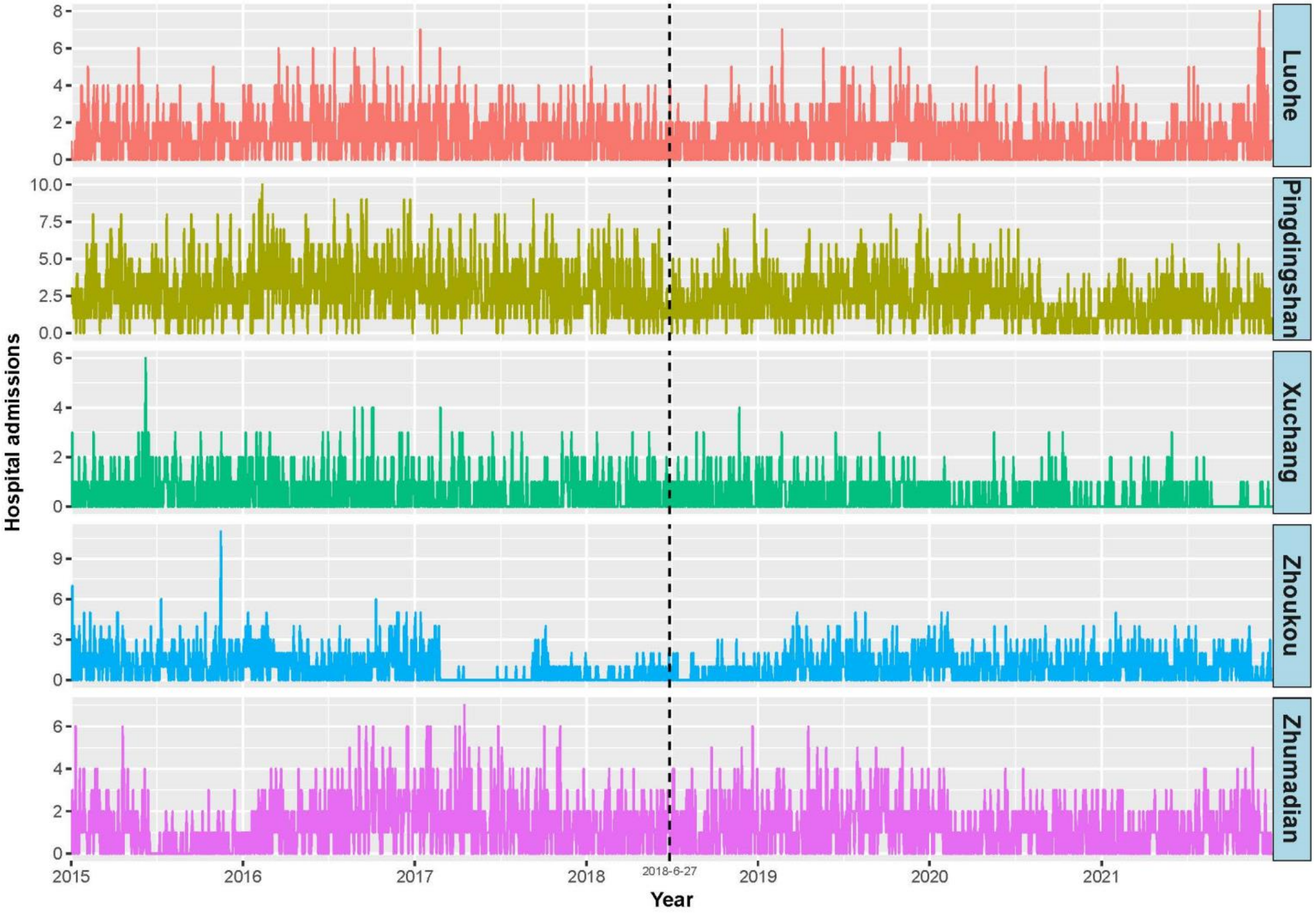
Luohe
Pingdingshan
Xuchang
Zhoukou
Zhumadian
Hospital admissions
Year
2018-6-27
2015
2016
2017
2018
2019
2020
2021

Figure S2.

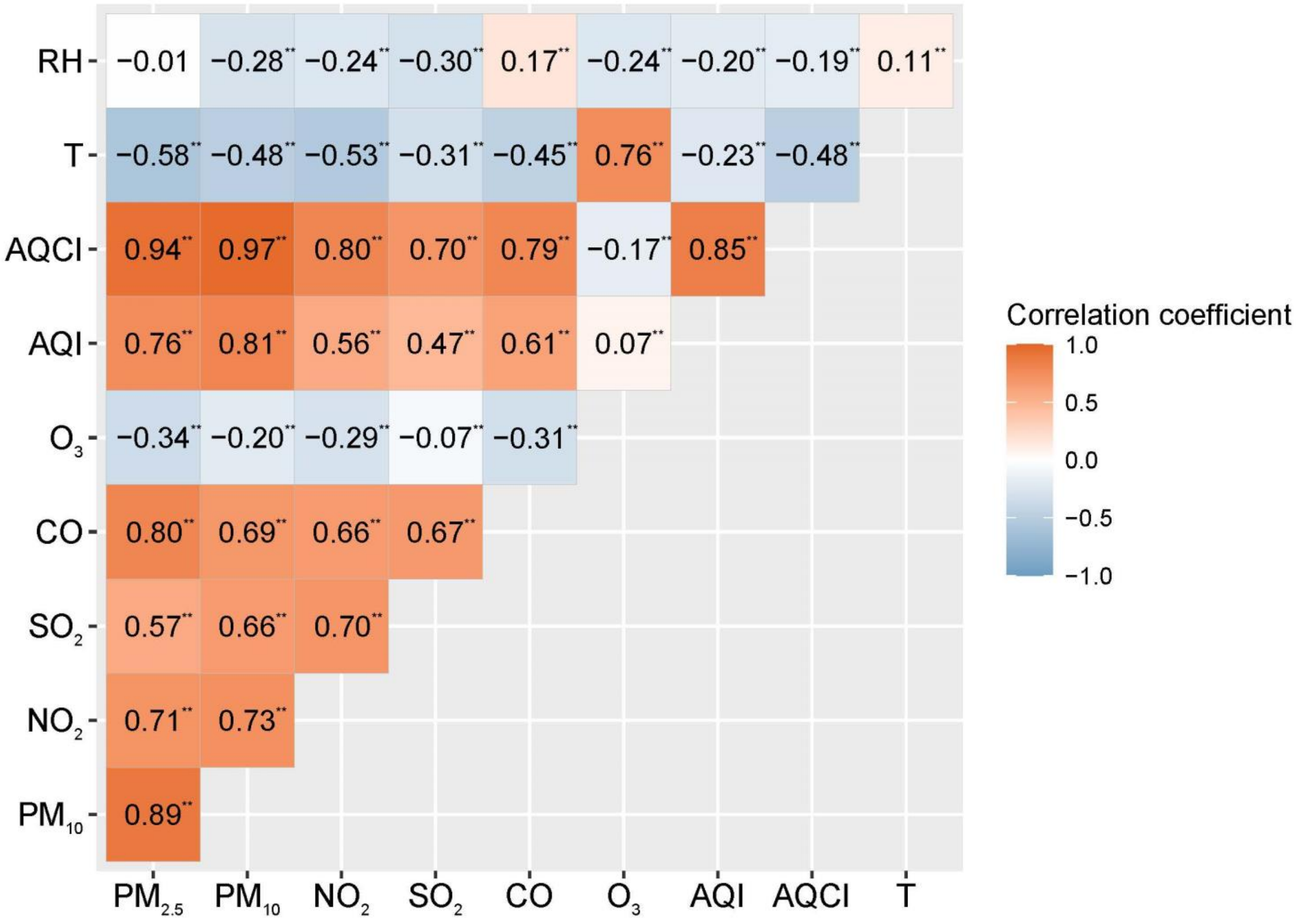
RH
T
AQCI
AQI
O_3
CO
SO_2
NO_2
PM_{10}
$PM_{2.5}$
PM_{10}
NO_2
SO_2
CO
O_3
AQI
AQCI
T
−0.01
−0.28**
−0.24**
−0.30**
0.17**
−0.24**
−0.20**
−0.19**
0.11**
−0.58**
−0.48**
−0.53**
−0.31**
−0.45**
0.76**
−0.23**
−0.48**
0.94**
0.97**
0.80**
0.70**
0.79**
−0.17**
0.85**
0.76**
0.81**
0.56**
0.47**
0.61**
0.07**
−0.34**
−0.20**
−0.29**
−0.07**
−0.31**
0.80**
0.69**
0.66**
0.67**
0.57**
0.66**
0.70**
0.71**
0.73**
0.89**
Correlation coefficient
1.0
0.5
0.0
−0.5
−1.0

Figure S3.

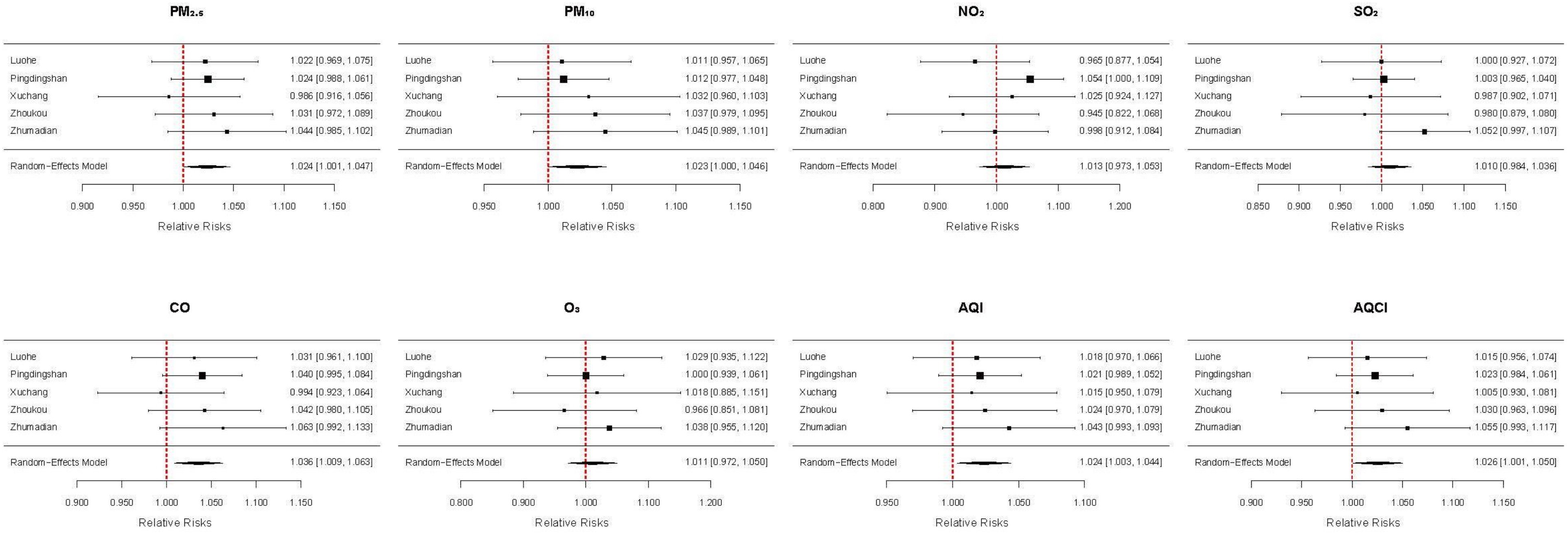
PM2.5
Luohe 1.022 [0.969, 1.075]
Pingdingshan 1.024 [0.988, 1.061]
Xuchang 0.986 [0.916, 1.056]
Zhoukou 1.031 [0.972, 1.089]
Zhumadian 1.044 [0.985, 1.102]
Random-Effects Model 1.024 [1.001, 1.047]
Relative Risks
PM10
Luohe 1.011 [0.957, 1.065]
Pingdingshan 1.012 [0.977, 1.048]
Xuchang 1.032 [0.960, 1.103]
Zhoukou 1.037 [0.979, 1.095]
Zhumadian 1.045 [0.989, 1.101]
Random-Effects Model 1.023 [1.000, 1.046]
Relative Risks
NO2
Luohe 0.965 [0.877, 1.054]
Pingdingshan 1.054 [1.000, 1.109]
Xuchang 1.025 [0.924, 1.127]
Zhoukou 0.945 [0.822, 1.068]
Zhumadian 0.998 [0.912, 1.084]
Random-Effects Model 1.013 [0.973, 1.053]
Relative Risks
SO2
Luohe 1.000 [0.927, 1.072]
Pingdingshan 1.003 [0.965, 1.040]
Xuchang 0.987 [0.902, 1.071]
Zhoukou 0.980 [0.879, 1.080]
Zhumadian 1.052 [0.997, 1.107]
Random-Effects Model 1.010 [0.984, 1.036]
Relative Risks
CO
Luohe 1.031 [0.961, 1.100]
Pingdingshan 1.040 [0.995, 1.084]
Xuchang 0.994 [0.923, 1.064]
Zhoukou 1.042 [0.980, 1.105]
Zhumadian 1.063 [0.992, 1.133]
Random-Effects Model 1.036 [1.009, 1.063]
Relative Risks
O3
Luohe 1.029 [0.935, 1.122]
Pingdingshan 1.000 [0.939, 1.061]
Xuchang 1.018 [0.885, 1.151]
Zhoukou 0.966 [0.851, 1.081]
Zhumadian 1.038 [0.955, 1.120]
Random-Effects Model 1.011 [0.972, 1.050]
Relative Risks
AQI
Luohe 1.018 [0.970, 1.066]
Pingdingshan 1.021 [0.989, 1.052]
Xuchang 1.015 [0.950, 1.079]
Zhoukou 1.024 [0.970, 1.079]
Zhumadian 1.043 [0.993, 1.093]
Random-Effects Model 1.024 [1.003, 1.044]
Relative Risks
AQCI
Luohe 1.015 [0.956, 1.074]
Pingdingshan 1.023 [0.984, 1.061]
Xuchang 1.005 [0.930, 1.081]
Zhoukou 1.030 [0.963, 1.096]
Zhumadian 1.055 [0.993, 1.117]
Random-Effects Model 1.026 [1.001, 1.050]
Relative Risks

Figure S4.

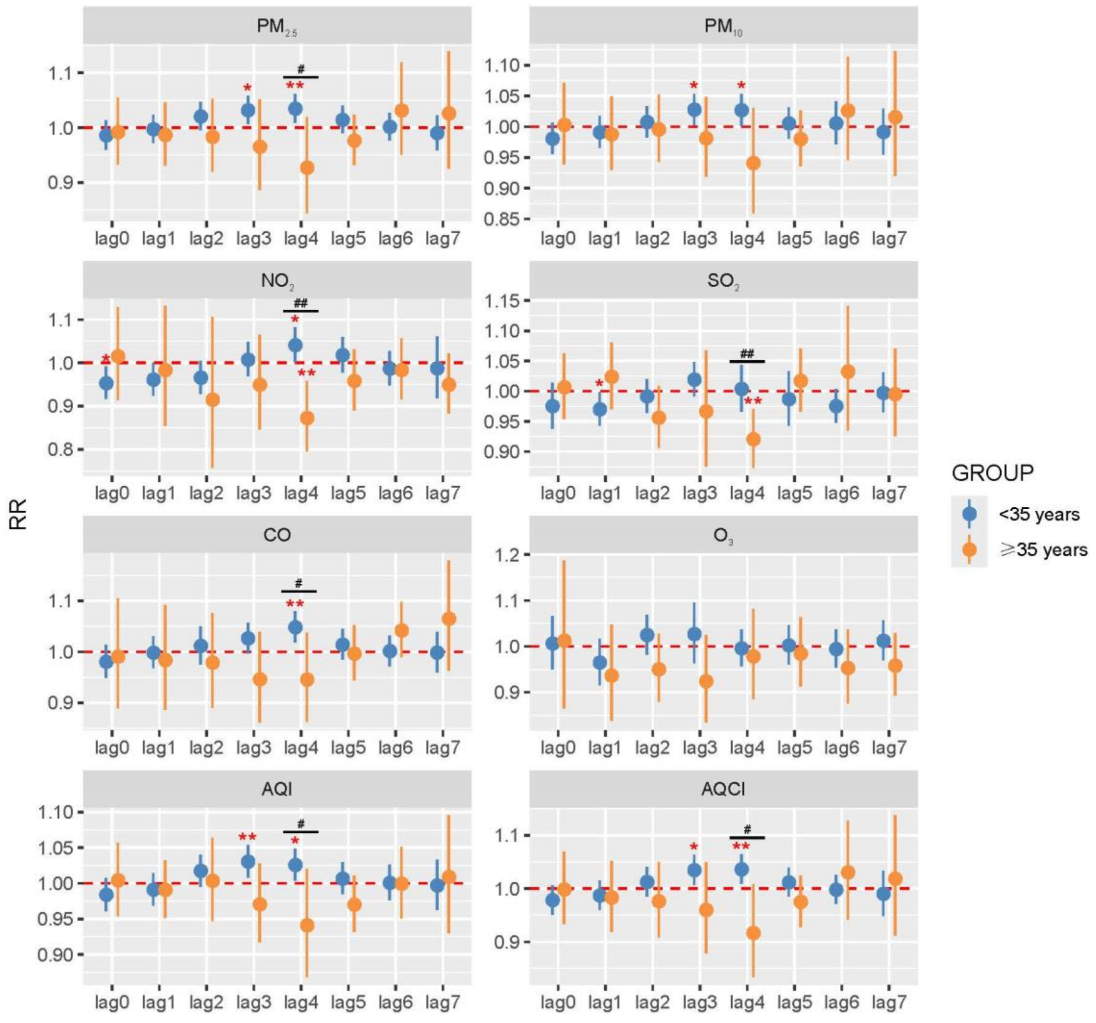

PM2.5
PM10
NO2
SO2
CO
O3
AQI
AQCI
RR
lag0 lag1 lag2 lag3 lag4 lag5 lag6 lag7
GROUP
<35 years
⩾35 years

Figure S5.

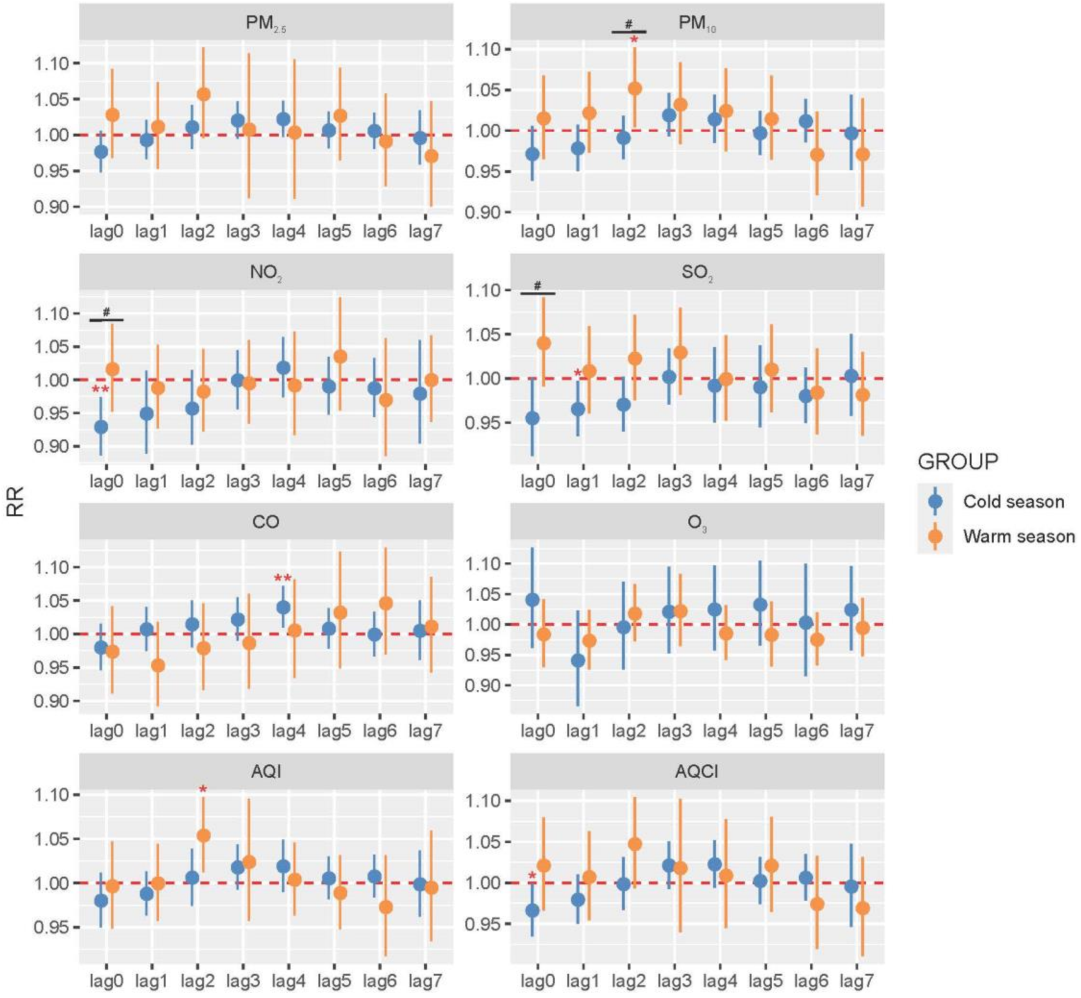
PM2.5
PM10
NO2
SO2
CO
O3
AQI
AQCI
RR
lag0 lag1 lag2 lag3 lag4 lag5 lag6 lag7
GROUP
Cold season
Warm season

Figure S6.

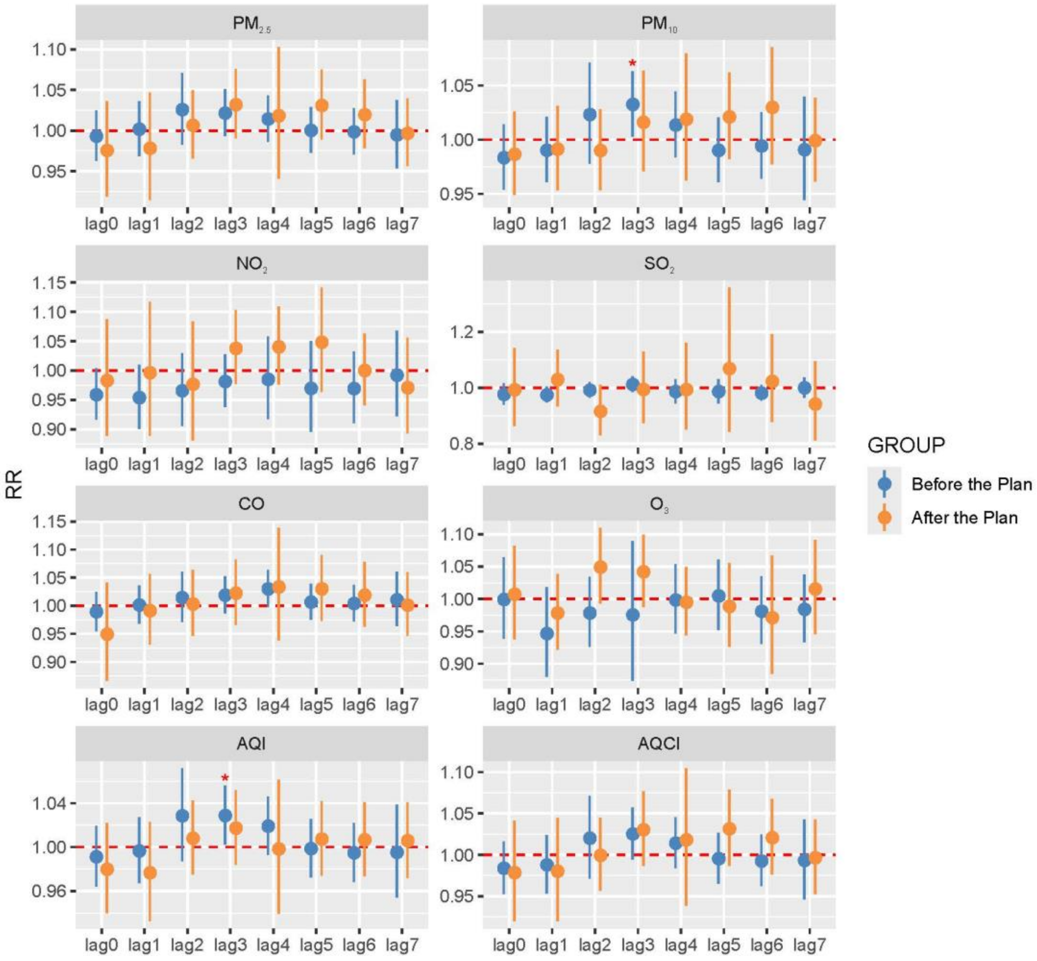

PM2.5
PM10
NO2
SO2
CO
O3
AQI
AQCI
RR
lag0 lag1 lag2 lag3 lag4 lag5 lag6 lag7
GROUP
Before the Plan
After the Plan

Graphical Abstract

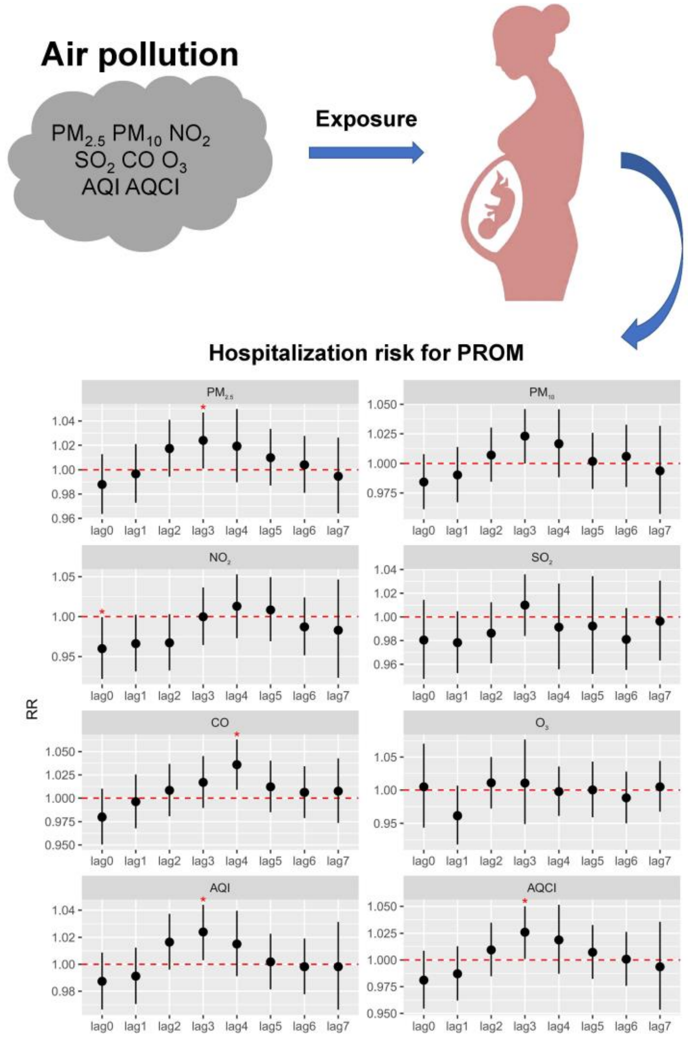

Supplement: Online Supplementary Document [file jogh-15-04165-s001.pdf]
